# Widespread Introduction of a High-Sensitivity Troponin Assay: Assessing the Impact on Patients and Health Services

**DOI:** 10.3390/jcm9061883

**Published:** 2020-06-16

**Authors:** Jaimi H. Greenslade, William Parsonage, Laura Foran, Louise McCormack, Sarah Ashover, Tanya Milburn, Sara Berndt, Martin Than, David Brain, Louise Cullen

**Affiliations:** 1 Emergency and Trauma Centre, Royal Brisbane and Women’s Hospital, Butterfield Street, Herston, QLD 4029, Australia; laura.foran@health.qld.gov.au; 2 Australian Centre for Health Services Innovation, School of Public Health and Social Work, Queensland University of Technology, 60 Musk Avenue, Kelvin Grove, QLD 4059, Australia; w.parsonage@me.com (W.P.); david.brain@qut.edu.au (D.B.); 3 Department of Cardiology, Royal Brisbane and Women’s Hospital, Butterfield Street, Herston, QLD 4029, Australia; 4 Healthcare improvement unit, Clinical Excellence Queensland, Butterfield Street, Herston, QLD 4006, Australia; Louise.McCormack2@health.qld.gov.au (L.M.); sarah.ashover@health.qld.gov.au (S.A.); tanya.milburn@health.qld.gov.au (T.M.); sara.berndt@health.qld.gov.au (S.B.); 5 Emergency Department, Christchurch Hospital, Riccarton Avenue, Christchurch Central City, Christchurch 8011, New Zealand; martin@thanstedman.onmicrosoft.com

**Keywords:** troponin, acute myocardial infarction, health services research

## Abstract

Adoption of High-sensitivity troponin (hs-cTn) assays by hospitals worldwide is increasing. We sought to determine the effects of a simultaneous state-wide hs-cTn assay introduction on the implementing health service. A quasi-experimental pre–post design was used. Participants included all adult patients presenting to 21 Australian hospitals who had troponin testing commenced within the Emergency Department (ED). Data were collected for 124,357 episodes of care between 30 April 2018 and 23 April 2019; six months pre- and six months post-implementation of the assay. The primary outcome was hospital length of stay (LOS). Secondary outcomes included ED LOS, 90-day cardiovascular mortality, elevated troponin, diagnosis of acute myocardial infarction (AMI), admission to a cardiology ward, invasive cardiac procedures, and total hospital costs. Following hs-cTn implementation, there was a 1.9-h (95% CI: −2.9 to −1.0 h) reduction in overall LOS. This equated to a cost saving of over 9 million Australian dollars per year. There was no increase in diagnosis of AMI, invasive cardiac procedures or ward admissions. The use of hs-cTn assays facilitates important benefits for health services by enabling more rapid evaluation protocols within the ED. This benefit may be considerable given the large cohort of emergency patients with possible ACS.

## 1. Introduction

Symptoms of chest pain, indicative of possible acute myocardial infarction (AMI), are one of the most common causes for emergency department (ED) admissions worldwide [1,2]. Patients with suspected acute coronary syndrome (ACS) account for approximately 10% of all emergency patients but only 5% of this cohort are ultimately diagnosed with AMI [3]. A cornerstone for the diagnosis of AMI is the finding of an elevated troponin value (values above the 99th percentile of a normal reference population [4]). This precipitates instigation of evidence-based therapies and hospital admission [4].

Over the past two decades, assays for the detection of troponin have advanced dramatically, now enabling the detection of low concentrations with high precision [5]. High-sensitivity cardiac troponin assays (hs-cTn) allow for more rapid determination of elevated troponin levels due to increased analytical and clinical sensitivity [4,6]. Accordingly, a key advantage is shortened timeframes for troponin testing [7,8]. The increased sensitivity of troponin assays could also potentially lead to improved patient outcomes through better targeting of therapies for coronary heart disease [9].

However, the potential benefits of hs-cTn assay introduction must be balanced with possible unintended harms. The use of hs-cTn assays have been linked to increased proportions of emergency patients with elevated troponin concentrations [9,10] and consequently may lead to overdiagnosis of AMI [11]. In addition, elevated troponin concentrations are seen in numerous clinical conditions other than AMI that are associated with myocardial injury (e.g., tachycardia, acute heart failure and sepsis) [4]. Although troponin values hold prognostic information for non-AMI conditions, clinical outcomes are rarely altered with this knowledge [12] and additional unnecessary investigations may occur [13]. Identification of additional patients with non-AMI related myocardial necrosis, therefore, may unnecessarily utilise hospital resources.

The objective of this study was to describe impacts on patient care and healthcare utilisation following the introduction of a hs-cTn assay (Beckman Coulter Access hsTnI) across a large health care service. Firstly, we examined ED and hospital length of stay (LOS) in the six months pre- and post-implementation of the hs-cTnI assay. Secondly, we reported the proportions of patients with elevated troponin values, admission to cardiology wards, invasive cardiac procedures, discharge diagnoses, 90-day mortality and costs in these two periods.

## 2. Experimental Section

### 2.1. Study Design and Setting

This is a pre–post comparison of consecutive patients who presented to EDs and underwent hs-cTn testing in 21 Queensland Health (QH) hospitals. QH provides public health care for 5 million people [14] with approximately 1.6 million emergency presentations per annum [15]. A single pathology provider is used across all public hospitals (Pathology Queensland). Data were collected between 30 April 2018 and 23 April 2019. The 21 QH hospitals had 24-h access to the laboratory-based hs-cTnI assay. Other smaller centers were not included as they utilised point-of-care assays. The Royal Brisbane and Women’s Hospital ethics committee waived the requirement for review (LNR/2018/QRBW/49723) as the project was a quality activity and not recognised as research according to the definition in the National Statement on Ethical Conduct in Human Research of the National Health and Medical Research Council.

### 2.2. Participants

Patients were included if they were 18 years or older and had one or more troponin tests ordered within four hours of ED presentation. Patients were excluded if they (1) discharged against medical advice, or (2) died during their admission. Such patients were excluded as the intended assessment or healthcare utilisation for such patients is unknown.

### 2.3. Intervention

The pre-intervention period was defined by the use of the Beckman Coulter AccuTnI+3 assay; an assay with the 99th percentile being 40 ng/L with the 10% coefficient of variation (CV) at 40 ng/L [16]. The post-intervention period began with the introduction of the Beckman Coulter Access hs-cTnI assay on the 25th of October 2018. Sex-specific 99th percentiles (10 ng/L for females and 20 ng/L for males) were recommended for use. The 10% CV for this assay is at 4 ng/L [17].

For both the pre- and post-intervention periods, patient management was at the discretion of the treating physician, but all sites had a recommended pathway based upon the Australian Heart Foundation and Cardiac Society of Australia and New Zealand guidelines [7]. In the pre-intervention period, the recommended cTn sampling interval was 6 to 8 h (after initial testing on presentation) for most patients. However, 10 sites had adopted the IMPACT protocol [18], which enabled 0 and 2/3 h sampling for low- to intermediate-risk patients. This was estimated to accelerate care for approximately one third of patients at sites where it was implemented [19].

In the post-implementation period following introduction of the new assay, a reduced sampling interval of two to three hours was adopted for all patients, regardless of risk. Existing pathways were modified to incorporate shorter testing intervals but all other elements of assessment (such as historical and examination findings) were unchanged. Online and in-house education and training for clinicians was supported by a designated project team.

### 2.4. Measurements

Data were obtained from several QH state-wide databases. Data on ED arrival and discharge time, demographics, discharge disposition and ED diagnosis were collated from the QH Emergency Data Collection (EDC). Troponin results were obtained from Auslab laboratory information system. Data on hospital discharge time, hospital admission, procedures, and hospital diagnosis were obtained from the Queensland Hospital Admitted Patient Data Collection (QHAPDC). Death data were obtained from the Queensland Death Registry. Deterministic linkage was used to link these three databases. This linkage method involves linking records based on exact agreement of selected match variables and is recommended where direct identifiers are available and are of good quality. Each database contains a unique patient record number (URN) that was used in combination with date and time data to link the patient records.

### 2.5. Outcomes

The primary outcome was hospital LOS, calculated as the difference between date and time of arrival and date and time of discharge as an acute care patient. Time spent in a healthcare facility as a nonacute patient was not incorporated in overall LOS (e.g., time in rehabilitation, geriatric or mental health wards). Secondary outcomes included ED LOS; 90-day cardiovascular mortality, defined as death resulting from an AMI, sudden cardiac death, heart failure, cardiovascular (CV) procedures, CV hemorrhage, or other CV causes; elevated troponin, defined as a troponin value > 99th percentile (40 ng/L using the Beckman’s AccuTnI+3 assay and >10 ng/L for females and >20 ng/L for males using the Beckman’s hs-cTn assay); ED and hospital diagnosis of AMI, defined using ICD-10 codes assigned on ED or hospital discharge (see Appendix A); admission to a cardiology ward; and invasive cardiac procedures, defined based on ICD-10-AM ACHI procedure codes (see Appendix A). We also calculated the cost-savings associated with the new assay, in comparison to the usual method. This provides decision-makers with information from an economic perspective, which is of value in the current decision-making climate, where health services that are both clinically and cost-effective, must be provided.

### 2.6. Data Analysis

A total of 1.6 million patients present to a Queensland ED each year [15] and approximately 6–7% of such patients are investigated for ACS. As such, we anticipated that approximately 50,000 patients would present to an ED in each study period. The average LOS in May 2018 was 45 h. Assuming similar standard deviation, 2000–2500 patients included per hospital, and a low intraclass correlation (0.01), this study was powered (>80%) to detect a LOS reduction by 4 h at a significance level of 0.05.

Data were analysed using Stata 14 (StataCorp, 2015, College Station, TX, USA). The cohort comprised episodes of care six months pre- and post- implementation of hs-cTn assay. Data for the two weeks after implementation of the hs-cTnI assay were not included as a mix of hs-cTnI and c-TnI were used during this time in some hospitals.

Descriptive statistics were calculated for the pre- and post-implementation periods. For all endpoints, data were presented for the overall cohort and by sex. For LOS, the median was reported for the pre- and post-implementation periods. The difference between the two medians and 95% confidence interval (CI) of that difference was calculated using quantile regression. Quantile regression was also conducted to adjust for a number of factors, including time (weeks after study commencement), triage category and arrival mode. Time was incorporated to ensure that the results were not confounded by changes in LOS that can occur across time due to nonintervention factors (e.g., seasonal changes). The median difference calculated after adjusting for time reflects the immediate change in LOS that occurred after implementation of the assay. Triage category and arrival mode were incorporated to adjust for the minor baseline differences in these variables across the two study periods.

The proportion of patients meeting the secondary endpoints of admission to a cardiac ward, invasive cardiac procedures, or elevated troponins were reported for the pre- and post- implementation periods. The difference in proportions and 95% CI of the difference were calculated using generalised linear models with a binomial distribution. As with the primary endpoint, all differences were then adjusted for time, triage category and arrival mode. For both the primary and secondary endpoints, CIs were adjusted for clustering of patients within hospitals.

For mortality, patients were followed up for 90 days after presentation to the ED. In cases where the patient re-presented within 90 days, a single representative admission was selected at random from within that period for inclusion in the study dataset. Kaplan-Meier survival functions were calculated for cardiovascular mortality.

To describe the potential savings associated with ward time, the difference in mean LOS between pre- and post-implementation was calculated. The average number of hours saved was multiplied by the hourly ward cost. We used previously published estimates for ward costs ($67 per hour) [3], but indexed these costs to 2020 Australian dollars using a commonly used cost conversion calculator [20]. This resulted in a cost per hour of $74.50.

## 3. Results

Data were available from 63,335 episodes in the pre- and 61,022 in the post-implementation periods. While the baseline data were similar across study periods, there were minor differences in triage category and arrival mode (Table 1).

**Figure 1:**
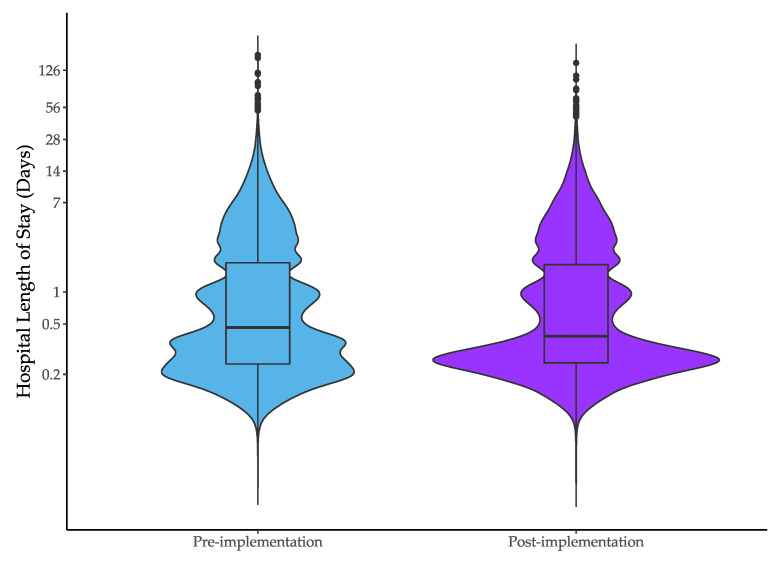
Hospital length of stay in the pre- and post-implementation periods. Data are presented on a logarithmic scale.

Median hospital LOS was 11.1 h (IQR 5.0–45.4) in the pre-intervention and 9.1 h (IQR = 5.1 to 43.7 h) in the post-intervention period; a reduction of 1.9 h (95% CI: −2.9 to −1.0 h, Table 2). There was a shift from LOS of 12–24 h during the pre-implementation period, to 4–12 h in the post-implementation period (Figure 1). After adjustment for time and baseline characteristics, the median difference was 0.7 h (95% CI: −1.3 to −0.1). Data for individual hospitals is provided in Appendix B and show a greater reduction in length of stay for hospitals where the IMPACT protocol had not been implemented in the pre-intervention period.

**Figure 2:**
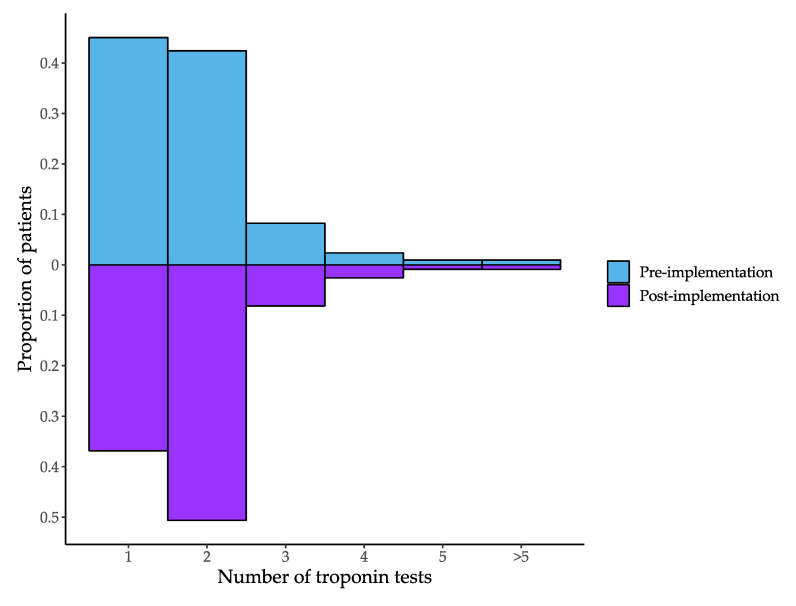
Number of troponin tests per episode in the pre- and post-implementation periods.

The proportion of episodes where only a single troponin was measured decreased from 46.7% pre-implementation to 37.6% post-implementation (Table 3). Patients had an average of 1.8 (SD = 1.0) troponin tests ordered in the pre- and 1.8 (SD = 0.9) in the post-implementation periods. There was a decrease in single troponin testing and an increase in dual troponin testing in the post-implementation period (Figure 2). The proportion of episodes where the patient had an elevated troponin increased from 12.5% pre-implementation to 20.9% post-implementation. The increase was larger for females (10.1% to 22.5%) than for males (14.9% to 19.4%).

There was no change in diagnosis of AMI with invasive cardiac procedures (coronary angiography, percutaneous coronary intervention, or coronary artery bypass grafting) (Table 4). There was a minor increase in admissions to any ward (73.1% to 74.3%) and a slight decrease in admissions to cardiology wards (6.8 to 5.7%). However, these differences did not emerge after adjustment for baseline characteristics. For those episodes where the patient did not have diagnosis of AMI, invasive cardiac procedures were performed in 947 (1.6%) pre-implementation and 874 (1.5%) post-implementation. For those episodes where the patient’s final diagnosis was AMI, invasive procedures were performed in 1593 (47.9%) pre-implementation and 1517 (46.9%) post implementation.

Death from a CV cause (up to 90 days after presentation) occurred in 513/54,600 (0.9%) pre-implementation patients and 333/52,310 (0.6%) post-implementation patients; a reduction of 0.3% (95% CI: −0.4 to −0.2). After adjustment for baseline characteristics, there was no difference in CV mortality in the two periods (−0.1%, 95% CI: −0.3 to 0.1%). Death from a CV cause occurred in 219 (0.8%) females pre- and 140 (0.5%) post-implementation. For males, CV deaths occurred in 294 (1.1%) patients pre- and 193 (0.7%) patient’s post-implementation.

There was no extra cost associated with using the new assay; the cost per test was the same for pre- and post-implementation periods, and the average number of troponin tests ordered per patient did not differ. Using unadjusted data, there was a 1.6-h difference in the mean length of stay between groups; a reduction in hospital costs of approximately $119.20 per person. Presuming 124,357 patients per year (as seen in this study), this would mean a saving of $14,823,354 per annum. Using adjusted data, there was a 1.0-h difference, making this saving $9,264,597. Adoption of the new assay would save the system 198,971 h of ward-bed time—approximately 8290 days—per annum, across the Queensland hospitals included.

## 4. Discussion

This is the first large study examining the implications of introducing the Beckman Coulter hs-cTnI assay into clinical practice. In this emergency population, implementing a hs-cTnI assay was associated with a moderate reduction in hospital LOS. There was an increased proportion of patients with troponin values above the 99th percentile but no increase in cardiac admissions, invasive coronary procedures or diagnosis of AMI.

The rate of detection of myocardial injury (with or without AMI) increased by 12% for females and 5% for males (approximately 8% overall) with the introduction of the hs-cTnI assay. The increase in females is higher than males as sites transitioned from using the overall 99th percentile cut point for the sensitive assay, to the new sex-specific 99th percentile cut points for the hs-cTn assay. The overall increase is slightly higher than that reported in previous research; there was an absolute increase of 4% using the Roche Elecsys hs-cTnT assay [10] and an increase of 3% using the Abbott Architect hs-cTnI [9]. The reason for differences compared with previous studies is unclear but may reflect differences in the different assays across studies.

Despite the increased identification of myocardial injury, diagnostic rates for AMI were unchanged. This finding is counter to previous research that has reported increases in diagnostic rates for AMI of between 4% and 5% after the introduction of the hs-cTnT assay [10,21]. Previous research was conducted based on the implementation of the hs-cTnT assay using data from patients presenting prior to 2013. It is possible that we did not find increased rates of AMI as there is a growing awareness that myocardial injury occurs in a number of conditions, and that diagnosis of AMI must be in the context of ischemia in addition to troponin values.

The proportion of patients undergoing single troponin testing decreased markedly following implementation of the hs-cTnI assay. This finding was not anticipated as there are some indications for single troponin testing when using a hs-cTnI assay but not a contemporary cTn assay [8]. During implementation, supporting education and documentation did not recommend the use of a single troponin testing strategy, due to limited data on the performance of limit of detection as a rule-out cutoff for this assay at the time. The reduction in single testing after introduction of the hs-cTn assay suggests improved compliance with guideline-based recommendations for serial sampling. The reduction of the timing of serial samples from 6 h when using a cTn assay to <3 h when using a hs-cTn assay may have increased the feasibility of serial sampling within a busy ED where there are time-based targets for admission or discharge [22].

Hospital LOS was reduced by 1−2 h in the current study. This is likely a conservative estimate of the LOS reductions that can be gained using a hs-cTn assay as around half of the sites had previously converted to the IMPACT pathway. This pathway incorporates two- to three- hour serial sampling using a sensitive troponin assay for patients with low- to intermediate-risk clinical features [18]. As shown in Appendix B, there tended to be larger reductions in LOS in sites where the IMPACT protocol had not been implemented. Prior research has yielded similar findings regarding reduced length of stay. The adoption of an ACS pathway utilising serial troponin testing within 3-h of presentation in New Zealand showed a reduction in LOS, an increase in patients discharged within six hours and no change in 30-day major adverse cardiac events [23]. A nonrandomised study of 2544 patients in six hospitals by the APACE group evaluated the impact of hs-cTnT on clinical care, and found a reduction in the LOS of 72 min for patients discharged from the ED, with no change in overall hospital LOS for admitted patients [10]. Given the large number of patients, this reduction in LOS is associated with substantial cost savings across the healthcare system. This represents a significant opportunity for capacity improvement in a system that is known to have access and throughput constraints.

The ED LOS was unchanged in our study. This may be due to the Australian focus on a 4-h disposition metric [22]. To meet this time target, the majority of hospitals have a process of completing initial testing within the ED setting before admitting patients to a short-stay or cardiac assessment unit to complete their assessment and/or await the results of their tests. As such, any LOS reductions gained by shorter serial sampling are reflected in the overall reduced hospital rather than the ED LOS.

Our study showed no change in the proportions of patients undergoing invasive cardiac procedures with the introduction of the new assay. Prior reports of the impact of hs-cTn assays on cardiac procedures is mixed. Some studies have shown no impact [10,24]. One study reported an absolute increase of angiography of approximately 3% with no change in the proportion of revascularisations [25], while another showed a 13% relative increase in angiography with an 18% increase in revascularisations [13].

This was based on administrative data. The accuracy of such data are not known, potentially influencing study estimates. In accordance with the universal definition of myocardial infarction, sex specific cut-points were reported for hs-cTn assay [4]. The specific impact of transition from overall to sex-specific cut-points cannot be determined. This study does not include any control hospitals (sites where the hs-cTn assay was not implemented) meaning that causality cannot be assumed. A stepped-wedge trial would have been of benefit in this regard, but this was not possible as the change to clinical care occurred on the same date across the entire health service. This study did include data on transfer to public hospitals within Queensland, transfers to private hospitals were not included. We are unable to report any data on noninvasive testing due to the lack of a standardised database across Queensland Health; however the APACE study found a reduction in stress testing [10]. We have not undertaken a full economic evaluation as part of this study and implementation costs have not been considered. However, the results still provide decision-makers with new and important information in relation to costs associated with adoption of the new assay. Further, in the economic analysis, we did not incorporate the cost of troponin testing as the mean number of troponin tests ordered did not differ in the pre- and post-implementation periods. However, our study did find that there was a reduction in the proportion of presentations where there was single troponin testing from 46.7% to 37.6%. Over a one-year period, this would equate to 11,317 presentations where there was an extra troponin test ordered. At the cost of $20.05 per test, this extra troponin testing would equate to $226,906. This may slightly reduce the cost benefits of implementing the assay.

## 5. Conclusions

The transition to a more precise troponin assay for patients with suspected ACS was associated with reduced hospital LOS, without an increase in cardiac admissions, invasive coronary procedures or diagnosis of AMI. The current study supports the increasing adoption of hs-cTn assays around the world. High sensitivity cTn assays enable more rapid evaluation protocols within the ED; a considerable benefit given the large cohort of emergency patients with possible ACS.

## Figures and Tables

**Table 1:** Baseline data during the pre- and post-implementation periods.

	Pre-Implementation (*n* = 63,335)	Post-Implementation (*n* = 61,022)
Mean age (SD)	61.0 (18.5)	59.6 (18.4)
Male sex, *n* (%)	32,287 (51.0%)	31,104 (51.0%)
Triage category, *n* (%)		
1—Immediate assessment and treatment	1325 (2.1%)	1204 (2.0%)
2—Assessment and treatment within 10 min	36,310 (57.3%)	36,205 (59.3%)
3—Assessment and treatment start within 30 min	23,347 (36.9%)	21,314 (34.9%)
4—Assessment and treatment start within 60 min	2289 (3.6%)	2229 (3.7%)
5—Assessment and treatment start within 120 min	63 (0.1%)	70 (0.1%)
Arrival by ambulance, *n* (%)	39,144 (61.8%)	37,419 (61.3%)

There were missing data for age (*n* = 10), sex (*n* = 5) and triage category (*n* = 1).

**Table 2:** Emergency Department (ED) and hospital length of stay.

	Pre-Implementation	Post-Implementation	Difference (95% CI)	Adjusted Difference * (95% CI)
**Overall Cohort**	***n*** **= 63,335**	***n*** **= 61,022**		
Median hospital length of stay (IQR), hours	11.1 (5.0 to 45.4)	9.1 (5.1 to 43.7)	**−1.9** **(−2.9 to −1.0)**	**−0.7** **(−1.3 to −0.1)**
Median ED length of stay (IQR), hours	4.0 (2.9 to 6.0)	4.0 (2.8–6.0)	0.0 (−0.1 to 0.2)	0.0 (−0.1 to 0.2)
Median hospital length of stay for patients managed in ED or short stay units (IQR), hours	8.6 (4.6–25.6)	7.4 (4.9–25.7)	**−1.2** **(−1.6 to −0.8)**	**−0.7** **(−1.1 to −0.2)**
**Females**	***n*** **= 31,048**	***n*** **= 29,913**		
Median hospital length of stay (IQR), hours	10.0 (4.9 to 38.8)	8.5 (5.1 to 38.1)	**−1.5** **(−2.3 to −0.8)**	−0.3 (−1.1 to 0.4)
**Males**	***n*** **= 32,287**	***n*** **= 31,104**		
Median hospital length of stay (IQR), hours	12.4 (5.1 to 50.2)	10.1 (5.1 to 47.7)	**−2.3** **(−3.5 to −1.2)**	**−1.1** **(−1.7 to −0.6)**

There were missing data for patient sex (*n* = 5). Bolded values represent differences where the confidence interval does not include 0. These are significant at *p* < 0.05. * Difference adjusted for time in weeks, triage category, and arrival mode.

**Table 3:** Troponin data.

	Pre-Implementation	Post-Implementation	Difference (95% CI)	Adjusted Difference * (95% CI)
**Overall cohort**	***n*** **= 63,335**	***n*** **= 61,022**		
Single troponin within 48 h	29,553 (46.7%)	22,958 (37.6%)	**−9.0%** **(−10.9 to −7.2%)**	**−7.4** **(−9.4 to −5.4%)**
Presentation Troponin >99th percentile	6970 (11.0%)	11,374 (18.7%)	**7.6** **(7.0 to 8.3%)**	**6.7%** **(5.8 to 7.6%)**
Any troponin within 12 h > 99th percentile	7919 (12.5%)	12,735 (20.9%)	**8.4%** **(7.7 to 9.0%)**	**7.5%** **(6.6 to 8.4%)**
**Females**	***n*** **= 31,048**	***n*** **= 29,913**		
Single troponin within 48 h	14,948 (48.1%)	11,252 (37.6%)	**−10.5%** **(−12.6 to −8.4%)**	**−9.4%** **(−11.8 to −7.0%)**
Presentation Troponin > 99^th^ percentile	2716 (8.8%)	6099 (20.4%)	**11.6%** **(10.7 to 12.6%)**	**10.7** **(9.7 to 11.8%)**
Any troponin within 12 h > 99th percentile	3120 (10.1%)	6711 (22.5%)	**12.4%** **(11.5−13.3%)**	**11.3%** **(10.3 to 12.2%)**
**Males**	***n*** **= 32,287**	***n*** **= 31,104**		
Single troponin within 48 h	14,605 (45.2%)	11,702 (37.6%)	**−7.6%** **(−9.3 to −5.9%)**	**−5.5%** **(−7.8 to −3.2%)**
Presentation Troponin > 99th percentile	4254 (13.2%)	5275 (17.0%)	**3.8%** **(3.0 to 4.5%)**	**2.7%** **(1.6 to 3.8%)**
Any troponin within 12 h > 99th percentile	4799 (14.9%)	6021 (19.4%)	**4.5%** **(3.8 to 5.2%)**	**3.6%** **(2.4 to 4.9%)**

There were missing data for presentation troponin value (*n* = 225), 12-h troponin value (*n* = 132), and sex (*n* = 5). Bolded values represent differences where the confidence interval does not include 0. These are significant at *p* < 0.05. * Difference adjusted for time in weeks, triage category, and arrival mode.

**Table 4:** Cardiac procedures and patient outcome.

	Pre-Implementation	Post-Implementation	Difference (95% CI)	Adjusted Difference * (95% CI)
**Overall cohort**	***n*** **= 63,335**	***n*** **= 61,022**		
ED diagnosis of AMI	685 (1.1%)	899 (1.5%)	**0.4%** **(0.1 to 0.7%)**	0.0% (−0.2 to 0.3%)
Hospital diagnosis of AMI	3328 (5.3%)	3232 (5.3%)	0.0% (−0.2 to 0.3%)	0.0% (−0.3 to 0.4%)
Admission to cardiology ward	4283 (6.8%)	3498 (5.7%)	**−1.0%** **(−1.8 to −0.3)**	0.0% (−0.8 to 0.8%)
Invasive cardiac procedures	2540 (4.0%)	2391 (3.9%)	−0.1% (−0.3 to 0.1%)	0.3% (−0.1 to 0.7%)
**Females**	***n*** **= 31,048**	***n*** **= 29,913**		
ED diagnosis of AMI	225 (0.7%)	350 (1.2%)	**0.4%** **(0.1 to 0.8%)**	0.1% (−0.1 to 0.3%)
Hospital diagnosis of AMI	1272 (4.1%)	1257 (4.2%)	0.1% (−0.3 to 0.5%)	0.4% (−0.1 to 0.8%)
Admission to cardiology ward	1662 (5.4%)	1378 (4.6%)	−0.7% (−1.5 to −0.0%)	0.2% (−0.9 to 1.2%)
Invasive cardiac procedures	861 (2.8%)	784 (2.6%)	−0.2% (−0.5 to 0.2%)	0.4% (−0.1 to 0.9%)
**Males**	***n*** **= 32,287**	***n*** **= 31,104**		
ED diagnosis of AMI	460 (1.4%)	549 (1.8%)	**0.3%** **(0.0 to 0.7%)**	−0.1% (−0.5 to 0.3%)
Hospital diagnosis of AMI	2056 (6.4%)	1975 (6.4%)	0.0% (−0.4 to 0.4%)	−0.4% (−1.1 to 0.3%)
Admission to cardiology ward	2621 (8.1%)	2120 (6.8%)	**−1.3%** **(−2.0 to −0.6)**	−0.2% (−1.0 to 0.7%)
Invasive cardiac procedures	1679 (5.2%)	1607 (5.2%)	0.0% (−0.3 to 0.3%)	0.1% (−0.6 to 0.8%)

Bolded values represent differences where the confidence interval does not include 0. These are significant at *p* < 0.05. * Difference adjusted for time in weeks, triage category, and arrival mode.

## Appendix A. Endpoint Codes

ED and hospital diagnosis of AMI were based on ICD-10 codes applied on discharge from ED or hospital respectively. The following codes were included I21, I21.0 121.1, I21.2, I21.3, I21.4, I21.9, I22, I22.0, I22.1, I22.8 or I22.9.

Invasive cardiac procedures were defined based on ICD-10-AM ACHI procedure codes of 38215-00, 38218-00, 38218-01, 38218-02, 38309-00, 38312-00, 38312-01, 38315-00, 38318-00, 38318-01, 90218-00, 90218-01, 90218-02, 90218-03, 38300-00, 38300-01, 38303-00, 38303-01, 38306-00, 38306-01, 38306-02, 38306-03, 38306-04, 38306-05, 38497-00, 38497-01, 38497-02, 38497-03, 38497-04, 38497-05, 38497-06, 38497-07, 38500-00, 38503-00, 38500-01, 38503-01, 38500-02, 38503-02, 38500-03, 38503-03, 38500-04, 38503-04, 38500-05, 38503-05, 90201-00, 90201-01, 90201-02, 90201-03, or 38637-00

## Appendix B

**Table A1:** Length of stay by hospital.

Site Number	Number of Patients	Pre-Intervention	Post-Intervention	Difference (95% CI)
1	6475	8.7 (5.1–41.8)	8.2 (5.2–48.5)	−0.4 (−1.0 to 0.1)
2	10,598	16.6 (4.9–46.9)	12.2 (5.4–41.6)	**−4.4 (−6.0 to −2.9)**
3	2022	22.0 (6.7–98.0)	7.6 (4.7–25.8)	**−14.4 (−18.8 to −10.0)**
4	8096	11.4 (6.8–43.5)	8.7 (5.9–33.3)	**−2.7 (−3.3 to −2.1)**
5	6946	21.2 (5.1–71.8)	21.6 (5.6–69.2)	0.4 (−2.0 to 2.7)
6	5020	13.7 (5.0–33.2)	7.8 (5.2–28.2)	**−5.9 (−7.4 to −4.5)**
7	6559	9.6 (5.3–28.8)	7.9 (5.0–28.5)	**−1.6 (−2.3 to −1.0)**
8	6390	11.0 (4.2–26.6)	6.8 (4.5–25.6)	**−4.2 (−5.4 to −3.1)**
9	11,339	11.7 (5.5–51.8)	9.9 (5.2–50.0)	**−1.8 (−3.1 to −0.6)**
10	4138	12.0 (4.3–38.8)	9.3 (4.8–32.4)	**−2.7 (−4.6 to −0.8)**
11	6151	6.8 (3.8–42.0)	6.7 (4.8–27.8)	−0.1 (−0.6 to 0.4)
12 *	5956	9.3 (5.0–33.0)	9.5 (4.9–42.5)	0.2 (−0.7 to 1.2)
13 *	7583	10.9 (4.3–47.0)	9.5 (4.6–47.0)	−1.4 (−3.2 to 0.3)
14 *	1244	6.9 (3.3–29.1)	6.9 (3.5–31.1)	0.1 (−1.7 to 1.8)
15 *	6958	9.4 (5.8–39.2)	8.3 (5.5–45.9)	**−1.1 (−1.6 to −0.6)**
16 *	4643	14.5 (5.1–62.0)	10.8 (5.2–47.9)	**−3.8 (−5.8 to −1.8)**
17 *	2765	9.2 (4.5–24.7)	7.6 (4.4–22.6)	**−1.7 (−2.9 to −0.5)**
18 *	6159	8.6 (5.2–36.2)	6.9 (5.1–41.7)	**−1.6 (−1.9 to −1.3)**
19 *	3944	15.0 (5.8–62.1)	12.2 (5.1–61.3)	**−2.8 (−5.6 to −0.2)**
20 *	4754	19.3 (5.3–79.9)	19.5 (5.1–79.2)	0.2 (−2.1 to 2.5)
21 *	6617	13.0 (4.9–45.0)	15.2 (5.4–45.7)	**2.3 (0.2 to 4.3)**

* Indicates a site where the IMPACT protocol was implemented prior to 2018. IMPACT allowed serial testing at 0- and 2/3-h for low- to intermediate-risk patients. In all other sites, 0- and 6-h troponin testing was recommended for all patients. Bolded values represent differences where the confidence interval does not include 0.
